# Zinc-energized dynamic hydrogel accelerates bone regeneration via potentiating the coupling of angiogenesis and osteogenesis

**DOI:** 10.3389/fbioe.2024.1389397

**Published:** 2024-04-03

**Authors:** Nanning Lv, Zhangzhe Zhou, Lihui Hong, Hongye Li, Mingming Liu, Zhonglai Qian

**Affiliations:** ^1^ Department of Orthopaedics, The First Affiliated Hospital of Soochow University, Soochow University, Suzhou, China; ^2^ Department of Orthopedic Surgery, The Affiliated Lianyungang Clinical College of Xuzhou Medical University (The Second People’s Hospital of Lianyungang), Lianyungang, China

**Keywords:** hydrogel, bioactive glass, zinc, angiogenesis, bone defect

## Abstract

Insufficient initial vascularization plays a pivotal role in the ineffectiveness of bone biomaterials for treating bone defects. Consequently, enhancing the angiogenic properties of bone repair biomaterials holds immense importance in augmenting the efficacy of bone regeneration. In this context, we have successfully engineered a composite hydrogel capable of promoting vascularization in the process of bone regeneration. To achieve this, the researchers first prepared an aminated bioactive glass containing zinc ions (AZnBg), and hyaluronic acid contains aldehyde groups (HA-CHO). The composite hydrogel was formed by combining AZnBg with gelatin methacryloyl (GelMA) and HA-CHO through Schiff base bonding. This composite hydrogel has good biocompatibility. In addition, the composite hydrogel exhibited significant osteoinductive activity, promoting the activity of ALP, the formation of calcium nodules, and the expression of osteogenic genes. Notably, the hydrogel also promoted umbilical vein endothelial cell migration as well as tube formation by releasing zinc ions. The results of *in vivo* study demonstrated that implantation of the composite hydrogel in the bone defect of the distal femur of rats could effectively stimulate bone generation and the development of new blood vessels, thus accelerating the bone healing process. In conclusion, the combining zinc-containing bioactive glass with hydrogels can effectively promote bone growth and angiogenesis, making it a viable option for the repair of critical-sized bone defects.

## 1 Introduction

Repair of bone defects caused by various pathologies such as severe trauma, tumor resection, infection and degenerative diseases has been a major clinical challenge, which also brings a huge economic burden to patients (Shi et al., 2021). Although bone tissue has a certain regenerative capacity, bone grafting is usually required to achieve effective treatment for large bone defects that exceed the self-healing capacity of bone tissue. Natural bone grafts, such as autologous and allogeneic bone grafts, are frequently employed for the treatment of bone defects, but they are associated with limitations, such as restricted availability, donor injury, immune rejection, and risk of infection (Zhou et al., 2023). More importantly, the number of traditionally available natural bone grafts is still far from meeting the clinical needs, especially in the current environment of increasing global aging and obesity epidemic. In recent years, the advancement of bone tissue engineering technology has emerged as a promising avenue for addressing bone defects. This approach holds potential for overcoming the challenges associated with bone regeneration (El-Rashidy et al., 2017; Zhang et al., 2022; Yoon et al., 2023).

Notably, the interplay between osteogenesis and angiogenesis plays a crucial role in the process of bone regeneration. Blood vessels, serving as an essential supplier of nutrients to bone tissue, not only facilitate the transportation of minerals and growth factors, thereby promoting calcium salt deposition in bone, but also release paracrine signals to regulate the growth, differentiation, and regeneration of various cell types (Zhang et al., 2020). When bone biomaterials are used to treat bone defects, the vascular network induced in the early stage can provide sufficient nutritional support for subsequent new bone tissue, and can effectively transport metabolic substances away (Simunovic and Finkenzeller, 2021). For example, in order to augment the vascularization of the collagen-hydroxyapatite (CHA) scaffold, the integration of alginate particles (MPS) coated with vascular endothelial growth factor (VEGF) was implemented into the CHA scaffold. The findings demonstrated that the composite scaffold exhibited the ability to steadily release bioactive VEGF, thereby effectively stimulating the development of blood vessels and the restoration of bone defects (Quinlan et al., 2017). Insufficient blood supply during the process of bone tissue regeneration poses challenges in repairing and rebuilding bone tissue defects. Consequently, the revascularization of bone biomaterials emerges as a critical determinant for the success of bone tissue regeneration. The development of bone biomaterials capable of promoting the regeneration of vascularized bone represents an efficacious approach for treating bone defects.

Hydrogel is an insoluble hydrophilic polymer that mimics the natural extracellular matrix and can transport nutrients and metabolites, and is widely used in tissue engineering (Ding et al., 2023). Currently, gelatin methacryloyl (GelMA), as an emerging biomaterial, has been widely used in the field of bone repair (Wang et al., 2023). GelMA is a new type of hydrogel for photochemical cross-linking obtained by modifying methacryloyl to gelatin, which better retains the arginine-glycine-aspartic acid sequence and matrix metalloproteinase binding sequence of gelatin. It can effectively enhance cell adhesion and differentiation and ensure its biodegradability, which has great potential for application in bone tissue engineering (Dong et al., 2019). In addition, due to the presence of methacrylic anhydride, GelMA is able to generate thermally stable cross-linked hydrogels by photoinitiators and ultraviolet (UV) irradiation. Therefore, aqueous solutions of GelMA can be injected into irregularly shaped bone defects and subsequently cured to meet the complexity and variety of bone defect shapes. However, the GelMA itself has limited biological properties such as angiogenesis and osteogenesis, as well as limited ability to control drug release (Xiao et al., 2019; Liu et al., 2020). To address these issues, the properties of GelMA hydrogels have been improved by incorporating chemical components such as nanomaterials, microspheres, and natural or synthetic polymers (Shao et al., 2019; Sakr et al., 2022).

In recent years, bioactive glass has been gradually favored due to its good cytocompatibility, osteoconductivity and osteoinductivity (El-Rashidy et al., 2017). After implantation into the site of bone defect, bioglass will react with body fluids and degrade slowly in the body while gradually releasing ions such as silicon, calcium and phosphorus, forming a hydroxyapatite layer on the surface of the scaffold, which is chemically close to the composition of human bone, and promoting osteoclast adhesion and bone regeneration (Kong et al., 2018). For example, Zhang et al. (2016) found that Sr-substituted submicron bioactive glasses modulate macrophage responses to improve bone regeneration. Furthermore, the adjustability of bioglass components allows for the incorporation of inorganic particles, thereby enhancing its biological properties. This process involves compounding additional functional particles and releasing trace elements that facilitate bone repair and tissue regeneration as the bioglass degrades. Ultimately, this mechanism can promote osteogenesis and vasculogenesis, expediting the process of bone repair (Zhao et al., 2019). For example, a ph-responsive nanoplatforms was constructed by *in situ* growth of zinc sulfide (ZnS) nanoparticles on the surface of Ti3C2 MXene nanosheets, which were then incorporated into poly-L-lactic acid (PLLA) to produce composite bone scaffolds. This scaffold exhibited effective biofilm clearance and osteogenic properties (Qian et al., 2024a).

Zinc is one of the many metal ions that are essential for bone formation and mineralization. It is mainly found in bone tissue (Huang et al., 2020; Molenda and Kolmas, 2023). Numerous studies have shown that zinc has improved mechanical properties, good biocompatibility and osteoinductive properties (Huang et al., 2021). Zinc ions can regulate the expression of osteogenesis-related genes, such as runt-related transcription factor 2 (Runx-2), collagen type I (COL1), alkaline phosphatase (ALP), osteocalcin (OCN) (Qi et al., 2020; Qu et al., 2020). In addition, Zinc can promote migration and differentiation of bone marrow mesenchyml stem cells (BMSCs) as well as angiogenesis by activating mitogen-activated protein kinase (MAPK) (Song et al., 2020). A wide range of concerns have been raised about the use of zinc to enhance the osteogenic and angiogenic properties of bioglass (Dittler et al., 2019; Zamani et al., 2019).

**FIGURE 1:**
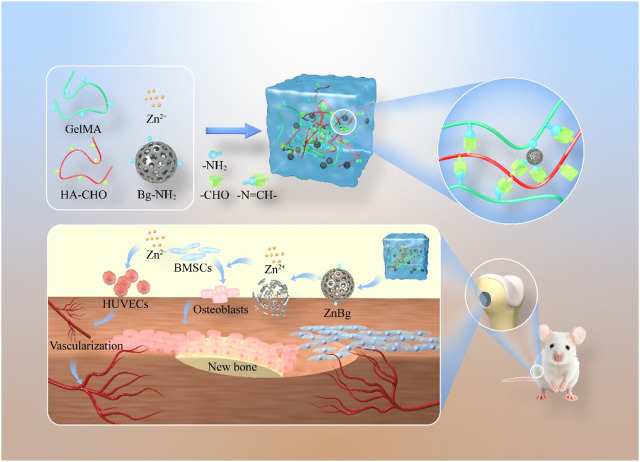
Schematic diagram of G/H@AZnBg promoting angiogenesis and enhancing osteogenesis.

In this study, we first prepared an aminated bioactive glass containing zinc ions, called AZnBg. AZnBg was then mixed in aldolylated hyaluronic acid (HA-CHO) and GelMA to form a composite hydrogel by photocross-linking of GelMA, termed G/H@AZnBg. During this process, the amino groups in AZnBg and GelMA formed Schiff bases with the aldehyde groups in HA-CHO, which maked the structure of the composite hydrogel more stable and could achieve acid-responsive ion release. In addition to characterizing the AZnBg particle morphology and release behavior, we also evaluated the *in vitro* angiogenic and osteoinductive activities of the composite hydrogel. Finally, the composite hydrogels were implanted into rat femoral defects to evaluate their ability to promote angiogenesis and bone formation *in vivo*. Figure 1 showed a schematic diagram of G/H@AZnBg promoting angiogenesis and promoting bone formation.

## 2 Materials and methods

### 2.1 Fabrication and characterization of materials

#### 2.1.1 Fabrication and characterization of AZnBg

3 g of silica nanoparticles was added into a beaker containing, 30 g of ethanol and 10 g of deionized water. The mixture was stirred at room temperature for 1 h after pH of mixture was adjusted to 6. Then a mixture of 0.3 g of KH550 and 5 g of ethanol was added to the silica solution. The white solution of dispersed silica nanoparticles was stirred at room temperature for 2 h. After reaction, the silica solution was separated by centrifugation and the precipitate of silica was washed three times with ethanol to remove unreacted KH550. Then 50 mL of ZnCl_2_ aqueous solution (0.2 M) was prepared by dissolving ZnCl_2_ in 50 mL of deionized water. The prepared silica nanoparticles (2 g) were added to zinc ions solution, stirred at room temperature for 2 h. After reaction, the mixture solution was separated by centrifugation and the precipitate of silica was washed three times with ethanol to remove excessive ZnCl_2_. The AZnBg was obtained and dried in a vacuum at 80°C for 12 h. The morphology of AZnBg was observed through transmission electron microscopy.

#### 2.1.2 Fabrication and characterization of HA-CHO

Mix 1.5 g HA with 150 mL deionized water. Add 802 mg sodium periodate and stir for 2 h. Stop the reaction by adding 200 μL ethylene glycol. Dialyze with deionized water. Freeze-dry the resulting HA-CHO and store at 4°C. ^1^H NMR spectra were recorded in D2O on a Bruker Avance 400 at 400 MHz. FTIR was used to detect the molecular structure of HA-CHO and HA.

#### 2.1.3 Fabrication of G/H@ AZnBg

A solution was prepared by dissolving 1 g of gelation, 100 mg of HA-CHO, and 50 mg of the photoinitiator lithium phenyl-2,4,6-trimethyl-benzoyl-phosphonic in 20 mL of deionized water at a temperature of 37°C. Subsequently, AZnBg was introduced into the aforementioned mixture. The resulting G/H@AZnBg composite was obtained through the process of photocrosslinking, utilizing a wavelength of 405 nm.

#### 2.1.4 Characterization of G/H@ AZnBg

The self-healing properties of the hydrogel were tested by rheological analysis. In short, oscillatory strain scans were conducted from 0.1% to 1000%. After allowing the hydrogel to heal for 20 min, an oscillating time scan was performed to characterize the self-healing effect. The self-healing ability was further demonstrated by cutting two different colors of hydrogels into two halves and reconnecting them after exchange. After allowing for healing for 15 min, display the reconnected sample. All rheological measurements were conducted at 25°C to minimize evaporation.

The freeze-dried hydrogel was immersed in 20 mL Tris-HCl buffer solution under shaking at 37°C, 10 mL buffer solution was collected at different predetermined time points, and the same volume of fresh buffer solution was used to supplement. The release profiles of Zn ions from the hydrogels in the buffer solution were measured by inductively coupled plasma atomic emission spectroscopy (ICP-AES, Varian 715 ES, California, USA).

The freeze-dried hydrogel was weighed (W_0_) and then immersed in 20 mL PBS buffer. The hydrogel was weighed after PBS was removed at the scheduled time (W_t_). The same amount of PBS is replaced after each measurement. The swelling rate of hydrogels was determined by the following equation:

$$\text{swelling}=\frac{\text{Wt}-W0}{W0}\;x100\%$$

Freeze-dried hydrogels are immersed in PBS buffers at 37°C (PBS buffers are replaced every 48 h). At the scheduled time, the hydrogel was taken out, washed with distilled water to remove excess PBS buffers, frozen in liquid nitrogen, and lyophilized. The weight of the initial and degraded hydrogels was recorded as W_0_ and W_d_, respectively.

$$\text{Residual weight}=\frac{\text{Wd}}{W0}x100\%$$

### 2.2 Biocompatibility and osteogenic effect of G/H@AZnBg

#### 2.2.1 Isolation and culture of BMSCs

8-week-old male Sprague-Dawley (SD) rat BMSCs were extracted with reference to previous studies (Zhou et al., 2023). Rat BMSCs were cultured using modified α-minimum medium (containing 10% fetal bovine serum, 1% penicillin, and streptomycin) at 37°C in an environment of 5% carbon dioxide. The medium was changed every 3 days, and third-generation BMSCs were used for the next study. A transwell’s co-culture system was used to assess the effect of G/H@AZnBg on osteogenesis. Hydrogels of different compositions (grouped as G/H, G/H@ABg, G/H@AZnBg) were immersed in the culture medium and placed in the upper chamber of the transwell for 12 h. BMSCs were inoculated and cultured in the lower layer of the transwell plate, and the medium was changed every 3 days.

#### 2.2.2 Cell viability assay and cell proliferation

BMSCs were co-cultured with hydrogels of different compositions (grouped as G/H, G/H@ABg, G/H@AZnBg) using transwell plates, and cell viability was assessed using a live/dead cell assay kit (Beyotime) at 1, 3, and 7 days, and finally, stained cells were photographed using a fluorescence microscope. In the same culture, cell proliferation was assessed using cell counting kit-8 (CCK-8, Beyotime) and absorbance at 450 nm was measured by spectrophotometer after 1, 3 and 7 days of culture.

#### 2.2.3 Scratch wound assay

BMSCs were first inoculated in 6-well plates cultured using conventional methods. When the cells were 90% full grown, a 200 µL sterile pipette tip was used to create a straight scratch in the cells, and then cell debris were washed off with PBS. Next the cells were cultured in conditioned medium containing different hydrogel compositions (grouped as G/H, G/H@ABg, G/H@AZnBg). Photographs of the scratch healing were taken at 6 and 12 h using a microscope.

### 2.3 Osteogenic effect of G/H@AZnBg

BMSCs were co-cultured with hydrogels of different compositions (grouped as G/H, G/H@ABg, G/H@AZnBg) using transwell plates, and the cell culture medium was replaced with osteoinductive medium. Alkaline phosphatase activity analysis was performed at day 7. Cells were stained with ALP chromogenic kit after fixation with 4% paraformaldehyde for half an hour. At day 14 and 21 of osteogenic induction, the cells were stained with alizarin red working solution after fixation with 4% paraformaldehyde. Images were taken with microscope.

RNA was extracted from the cell samples using Trizol reagent, and then reverse transcribed into cDNA using a reverse transcription kit. Finally, real-time fluorescence quantitative polymerase chain reaction (RT-qPCR) was applied to examinated the expression of four osteogenesis-related genes (*Col1a1*, *Runx2*, *ALP*, and *OCN*), and the internal reference gene was GAPDH.

### 2.4 Angiogenic effect of G/H@AZnBg

Growth factor-reduced Matrigel was first dropped into 24-well plates according to the instructions, and HUVECs were inoculated into 24-well plates after incubation at 37°C for 30 min. Then the HUVECs were incubated with medium containing different hydrogel compositions (grouped as G/H, G/H@ABg, G/H@AZnBg). 8 h later, the blood vessel formation was observed and photographed. Finally, the blood vessel formation was quantitatively analyzed using ImageJ software.

The steps about the scratch wound assay of HUVECs were the same as that of BMSCs. Briefly, we created scratches in the cells first, and then stimulated them with different hydrogel extracts and observed the migration of the cells at 6 and 12 h. In addition, we also used crystal violet staining to further verify the effect of hydrogels on the migration ability of HUVECs.

For immunofluorescence staining, HUVECs were stimulated by different hydrogel extracts, incubated with CD31 antibody and treated with fluorescent secondary antibody and DAPI. Then fluorescence images were taken, and the immunofluorescence intensity was analyzed semi-quantitatively by ImageJ.

### 2.5 *In vivo* evaluation of G/H@AZnBg on bone regeneration

All animal experimental protocols in this study were approved by the Ethics Committee of Soochow University. 8-week-old male SD rats weighing about 250 g were selected to establish a rat cranial critical size defect model. Four groups were randomly divided into (1) Defect group, (2) G/H group, (3) G/H@ABg, and (4) G/H@AZnBg. Sterilized hydrogel was injected into the cranial defect and then the tissue was sutured. The rats were executed at 4 and 8 weeks postoperatively, and the skulls were removed and fixed with 4% paraformaldehyde for 48 h. The skulls were scanned using Micro-CT, and three-dimensional reconstruction of regenerated bone tissues at the defects and quantitative analysis of trabecular number (Tb.N, 1/mm), bone tissue volume/total tissue volume (BV/TV) and bone mineral density (BMD, g/cm^3^) in the region of interest (ROI) were performed using NRecon v1.6 and CTAn v113.8.1 software. The region of interest (ROI) was defined as 3 mm in diameter and 2 mm in height. The skulls were next decalcified by immersion in EDTA for 1 month, and then the specimens were paraffin-embedded and histologically sectioned (5 μm thickness). Hematoxylin-Eosin staining (H&E) was performed to assess new bone formation according to the instructions.

Immunohistochemical staining for the bone formation-associated protein COLIA1 and the vascularization-associated protein VEGFA was performed to further reveal new bone and blood vessel formation. First, sections were incubated with 3% hydrogen peroxide to block endogenous peroxidase activity, followed by antigen repair for 30 min. next, sections were incubated in COLIA1 (1:500) and VEGFA (1:500) primary antibodies at low temperature overnight. Staining was then performed with the HRP-DAB kit according to the instructions. Finally, the sections were immersed in hematoxylin solution to stain the nuclei, dehydrated and sealed, and images were taken with a microscope.

### 2.6 Statistical analysis

All data were expressed as Mean ± SD. Comparison between two groups was performed by unpaired *t*-test (two-tailed), and comparison between multiple groups was performed by one-way analysis of variance or two-way analysis of variance combined with Tukey multiple comparisons. (*) *p*<0.05 was considered to indicate statistical significance.

## 3 Results

### 3.1 Characterization of G/H@AZnBg

**FIGURE 2:**
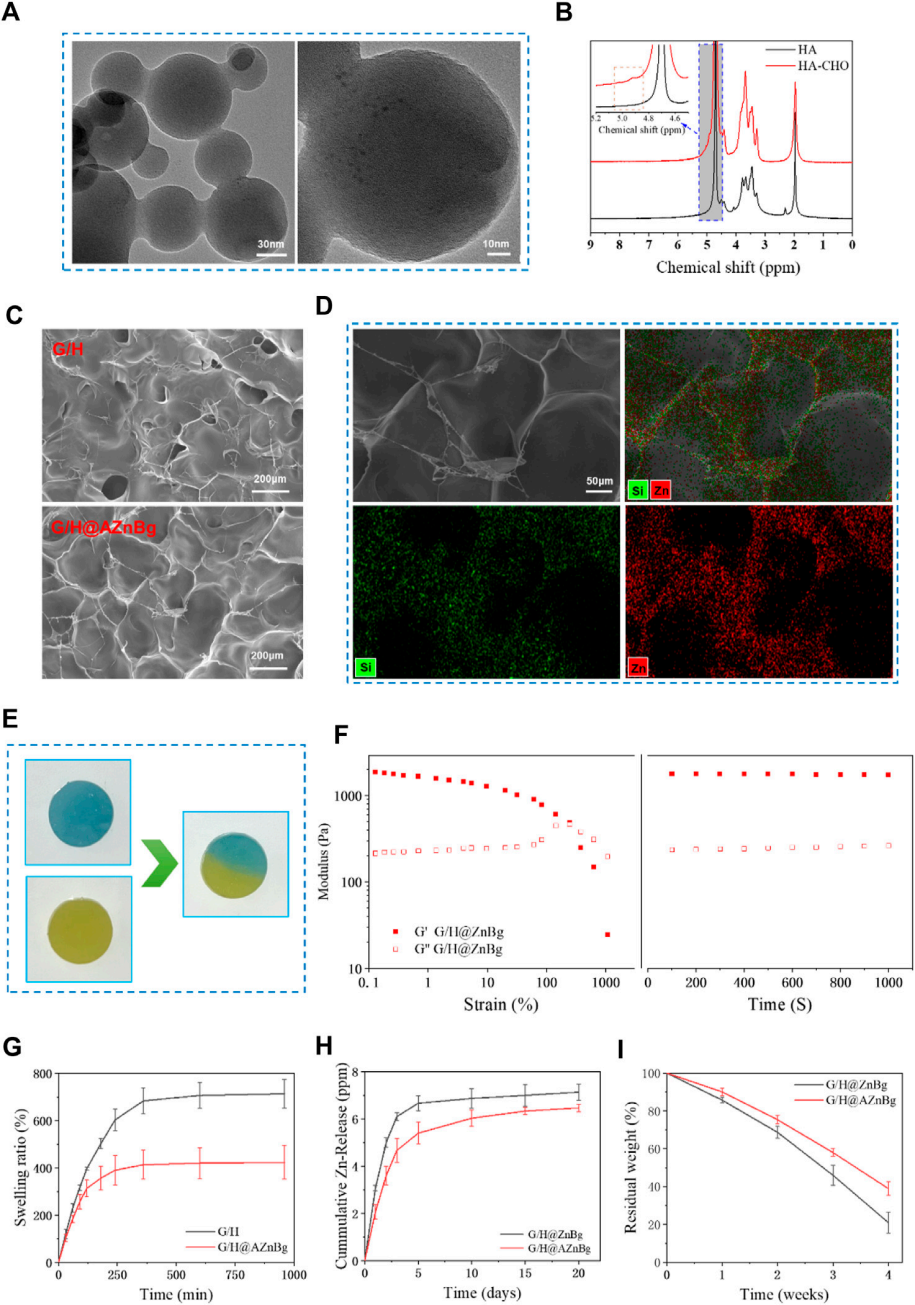
Characterization of materials. **(A)** TEM images of AZnBg. **(B)** 1H-NMR spectrum of HA and HA-CHO. **(C)** SEM images of G/H and G/H@AZnBg. **(D)** SEM images and element mapping (Si, Zn) of G/H@AZnBg. **(E)** Self-healing images of G/H@AZnBg. **(F)** Rheological measurements of G/H@AZnBg. **(G)** Swelling ratio of G/H and G/H@AZnBg. **(H)** Release of Zn ions from G/H@ZnBg and G/H@AZnBg. **(I)** Degradation of G/H@ZnBg and G/H@AZnBg.

Transmission electron microscope (TEM) showed that bioglass had a spherical structure of nanometer size (Figure 2A). As shown in Figure 2B, two new peaks at 4.9 ppm and 5.0 ppm were observed on the 1H-NMR spectrum of HA-CHO. This may correspond to hemiacetalic protons formed from the aldehyde groups and neighboring hydroxyl groups. In addition, FTIR showed that compared with HA, a new peak was observed at 1720cm^-1^, which was ascribed to the stretching vibration of the novel aldehyde group of HA-CHO (Supplementary Figure S1). Then, the aldehyde group peak vanished at 1,720 cm^−1^ and new peaks manifested at 1,670 cm^−1^, offering strong evidence that the aldehyde group of HA-CHO responds with the amino group of G/H@AZnBg to generate a C=N bond (Supplementary Figure S1). Scanning electron microscope (SEM) showed that hydrogels had a good three-dimensional network structure, which facilitated cell migration and nutrient transport (Figure 2C). Figure 2D showed the element mapping of Si and Zn ion in G/H@AZnBg. From the element mapping images, it could be found that the AZnBg were evenly distributed in the hydrogel.

In order to verify the self-healing ability, two circular hydrogels of different colors were cut, alternately placed and contacted for self-healing. As shown in Figure 2E, at 37°C, the hydrogel contact interfaces are fused together without cracks. When the strain increased from 0.1% to 10%, the hydrogel’s G′ and G″ values remained stable in the Figure 2F. Further increasing the strain to 1000% resulted in a decrease in G′ value, but an increase in G″ value followed by the appearance of a crossover point. In the next 1000 s, under the condition of 1% frequency and 1% strain, the internal structure of the hydrogel remained stable with the increase of time.

The swelling characteristics of hydrogels reflected their stability in humid environments (Figure 2G). In phosphate buffered saline solution at 37°C and pH 7.4, G/H@AZnBg first reacheed swelling equilibrium, with an equilibrium value of about 400%, while the equilibrium value of G/H was about 700%. After complete swelling, the hydrogel still retained the complete structure.

The release curve of zinc ion in hydrogel was evaluated by inductively coupled plasma atomic emission spectrometry (ICP-AES). The cumulative release of zinc ions in the hydrogel was shown in Figure 2H. In the first 5 days, the concentration of zinc ions significantly increased, and then the upward trend remained slow and stable. In addition, compared with G/H@ZnBg, the release rate of zinc ions in G/H@AZnBg was slower, which may be related to the interaction between amino modified bioglass and hydrogel, resulting in the change of network structure. Similarly, the release rate of Si ions in G/H@AZnBg were lower than that in G/H@ZnBg (Supplementary Figure S2). On the other hand, we have also found that zinc ions in hydrogels were released faster in acidic environments (Supplementary Figure S3).

The degradability of hydrogel is an important reference factor for *in vivo* application (Figure 2I). The weight of both hydrogels gradually decreased with time. The degradation rate of G/H-AZnBg is slower than that of G/H-ZnBg, which indicates that the interaction between amino-modified bioglass and hydrogel is stronger.

### 3.2 Biocompatibility of G/H@AZnBg

**FIGURE 3:**
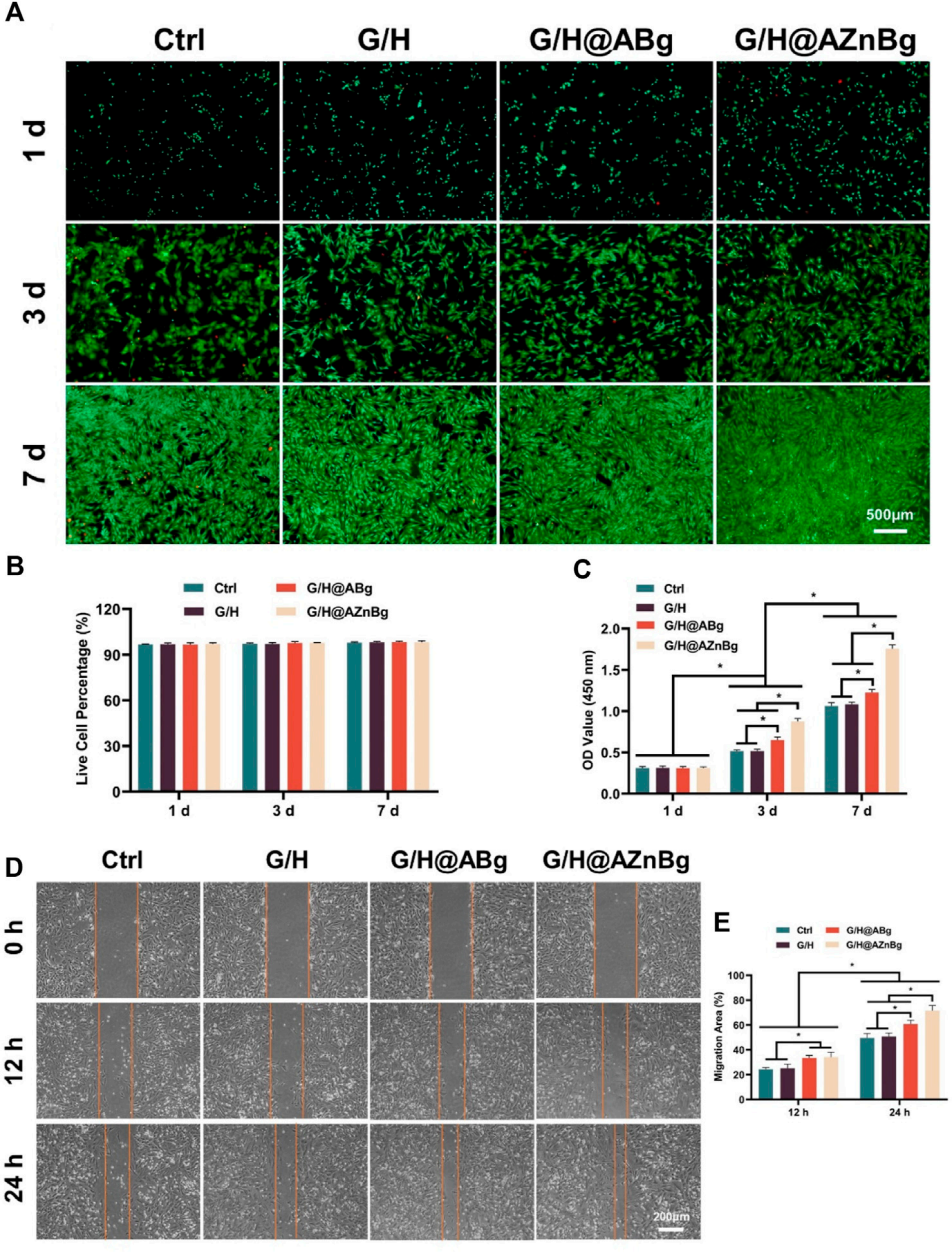
Biocompatibility of G/H@AZnBg. **(A)** Live/dead staining of BMSCs. **(B)** Quantitative analysis of live cell percentage of BMSCs. **(C)** CCK-8 assay indicating the proliferation rate of BMSCs after 1, 3 and 7 days. **(D)** Cell scratch assay of BMSCs at 0, 12, and 24 h. **(E)** Quantitative analysis of migration area.

The biocompatibility of the hydrogels was assessed by examining the effects of cell viability, proliferation, and migration ability. As shown in Figure 3A, live/dead cell staining of all groups showed a large number of live cells and very few dead cells. Quantitative analysis further confirmed that the proportion of live cells was similar in the groups, indicating no cytotoxicity to BMSCs (Figure 3B). The results of CCK-8 showed that on the third and fifth day, the cell proliferation was faster in the G/H@AZnBg and G/H@ABg groups compared with the Ctrl and G/H groups (Figure 3C). G/H@AZnBg had the strongest cell proliferation capacity. In addition, the results of cell scratch assay showed that the G/H@AZnBg and G/H@ABg groups promoted cell migration faster than the other groups at 12h and 24h, and the G/H@AZnBg group was the strongest in cell migration ability (Figures 3D, E).

### 3.3 *In vitro* evaluation of osteogenesis

**FIGURE 4:**
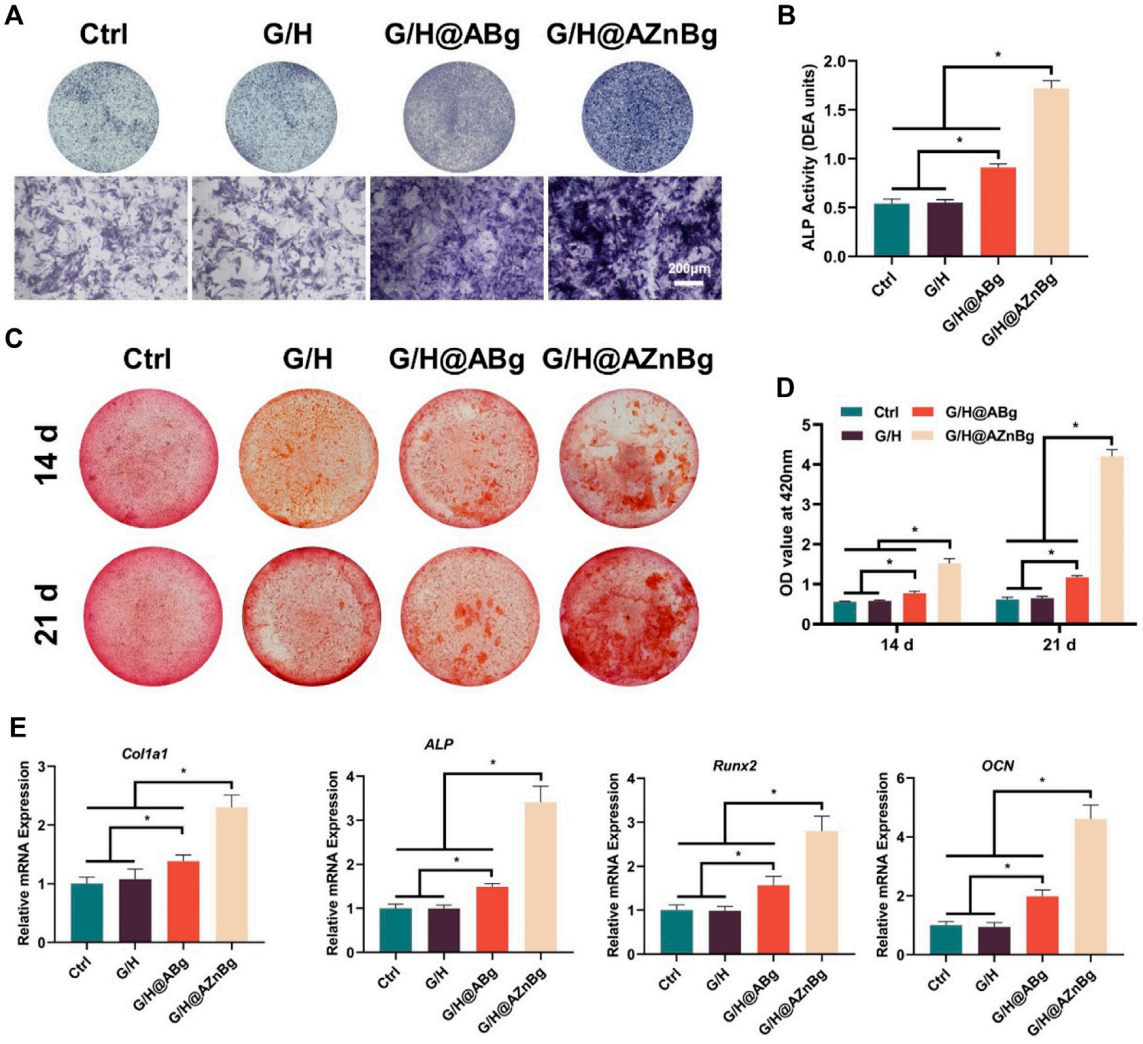
Effect of G/H@AZnBg on the osteogenic differentiation of BMSCs. **(A)** ALP staining after 7 days of induction. **(B)** Quantitative analysis of ALP activity. **(C)** Alizarin Red S (ARS) staining after 14 and 21 days. **(D)** Quantification of the stained calcium nodule. **(E)** The gene expression of osteogenic makers, including *Col1a1, ALP, Runx2, and OCN.* Data are presented as means ± SEM. Statistically significant differences were indicated by *p < 0.05.

We evaluated the effect of G/H@AZnBg on the osteogenic potential of BMSCs. G/H@AZnBg showed the highest ALP activity (Figure 4A). Quantitative analysis showed higher expression of ALP activity in the G/H@AZnBg and G/H@ABg groups compared to the Ctrl and G/H groups (Figure 4B). Calcium nodules are late markers of differentiation of BMSCs. After 14 and 21 days of osteogenic induction, a large number of positive calcium nodules were observed in the G/H@AZnBg and G/H@ABg groups (Figure 4C). Quantitative analysis showed that at 21 days, the degree of matrix mineralization in G/H@AZnBg was 5.5 and 2.6 times higher than that in G/H and G/H@ABg, respectively (Figure 4D).

In addition, PCR results showed that the G/H@AZnBg and G/H@ABg groups promoted the expression of osteogenesis-related genes (*Col1a1*, *ALP*, *Runx2*, *OCN*) in BMSCs (Figure 4E). The G/H@AZnBg group showed the strongest mRNA expression of ALP compared with the other groups (Figure 4E). These findings suggested that G/H@AZnBg effectively promoted osteogenic differentiation of BMSCs by enhancing gene expression of matrix mineralization and osteogenesis-specific markers.

### 3.4 *In vitro* evaluation of angiogenesis

**FIGURE 5:**
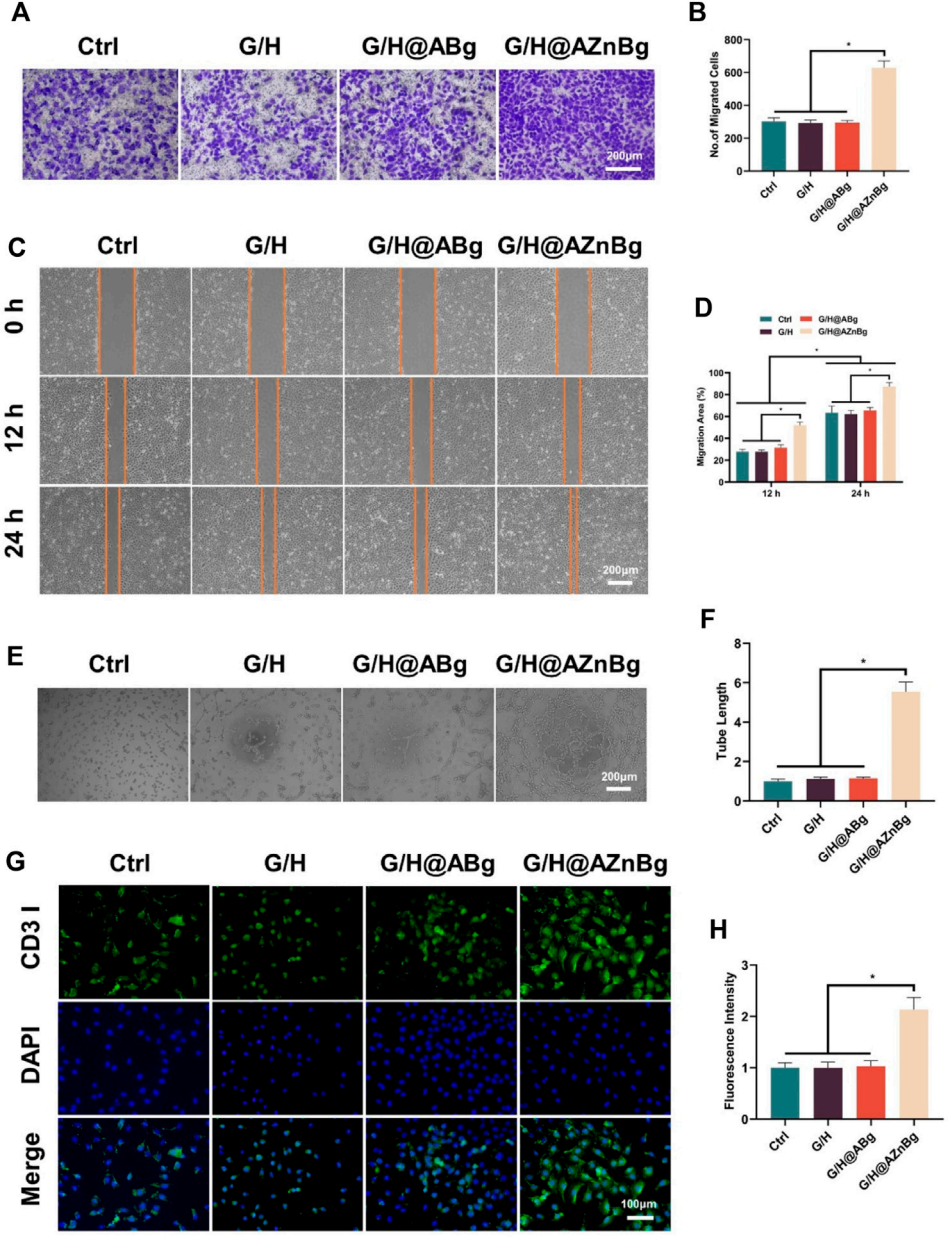
Effect of G/H@AZnBg on angiogenesis of HUVECs. **(A)** Positive migrating cells stained with crystal violet. **(B)** Quantitative analysis of migrating cells stained with crystal violet. **(C)** Cell scratch assay of HUVECs at 0, 12, and 24 h. **(D)** Quantitative analysis of migration area. **(E)** Tube formation assay. **(F)** Quantitative analysis of tube length. **(G)** Immunofluorescence staining of CD31. **(H)** Fluorescence intensity of CD31. Data are presented as means ± SEM. Statistically significant differences were indicated by *p < 0.05.

Early angiogenesis is a critical factor for successful bone repair. We evaluated the effect of G/H@AZnBg on vascularization by cell migration assay, angiogenesis, and immunofluorescence staining assay. Result of crystal violet staining showed that the number of positive migrating cells in G/H@AZnBg group was the highest (Figures 5A, B). Then, the cell scratch assay showed that the healing area in the G/H@AZnBg group was the largest and had the strongest migratory ability at 12h and 24h compared to the other groups (Figures 5C, D). These results indicated that G/H@AZnBg promoted the migration of HUVECs. In addition, angiogenic capacity of G/H@AZnBg was assessed by tubule formation assay. Compared with the other three groups, we observed that the G/H@AZnBg group allowed HUVECs to aggregate and build primary vessel-like network structures, whereas the other groups did not form (Figure 5E). Quantitative analysis also showed that the G/H@AZnBg group had the longest length of tube formation (Figure 5F). CD31 is an important endothelial marker for vascularization. The results obtained from immunofluorescence staining and subsequent quantitative analysis of fluorescence intensity indicated a statistically significant increase in the expression of CD31 in the G/H@AZnBg group when compared to the other groups (Figures 5G, H). These results indicated that G/H@AZnBg could promote angiogenesis.

### 3.5 *In vivo* bone repair assessment

**FIGURE 6:**
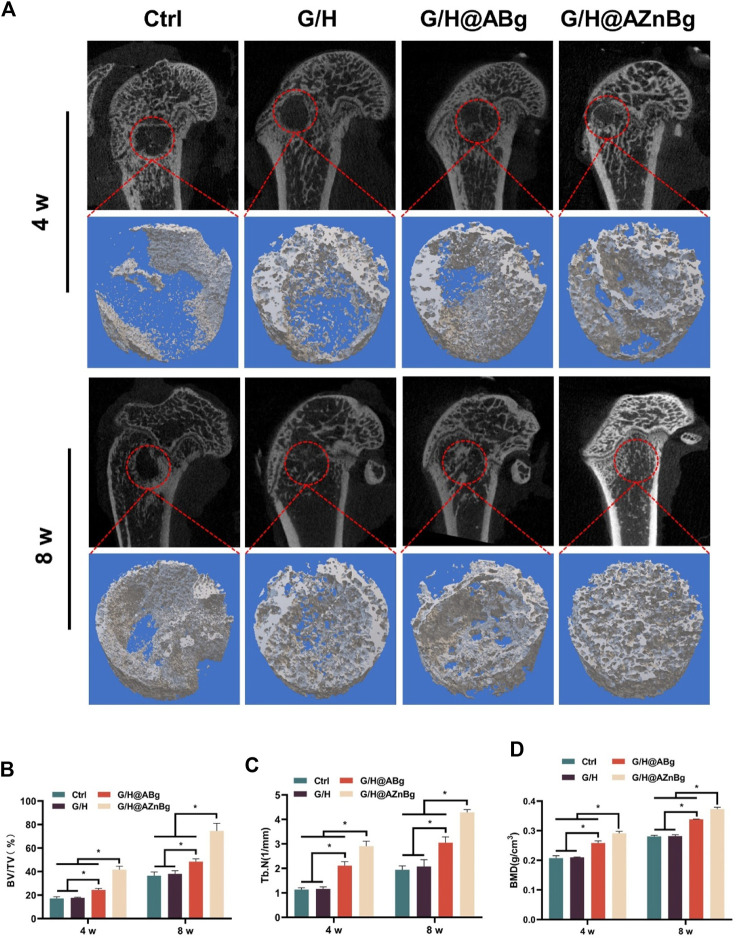
Micro-CT evaluation of *in vivo* bone defect regeneration. **(A)** Micro-CT imaging and 3D reconstruction of defect areas at 4 and 8 weeks. **(B–D)** Quantitative analysis of BV/TV, Tb.N and BMD in defect areas. Data are presented as means ± SEM. Statistically significant differences were indicated by *p < 0.05.

**FIGURE 7:**
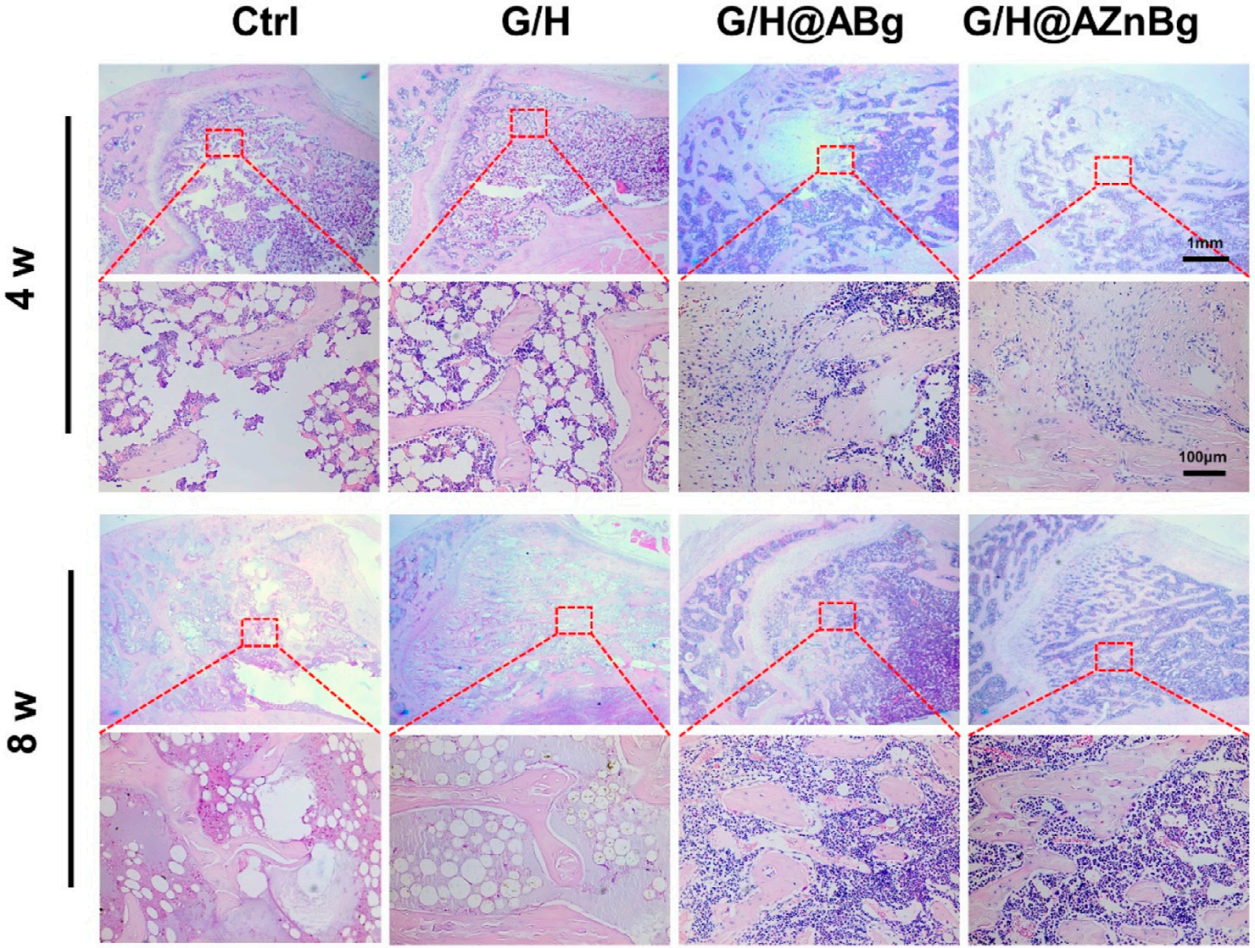
Histological analysis of the newly formed bone tissue. Representative hematoxylin and eosin (H&E) staining of the defect area implanted with G/H@AZnBg at 4 and 8 weeks post-surgery.

Composite hydrogels were implanted into the femoral defects of rats to observe bone formation. In the Ctrl and G/H groups, almost no bone formation was observed 4 weeks after surgery (Figure 6A). Some bone tissue was found at the femoral defect in the G/H@ABg group and new bone formation was seen in the G/H@AZnBg group (Figure 6A). Quantitative analysis showed the BV/TV in the G/H@AZnBg reached 41.6% at the fourth postoperative week (Figure 6B). In addition, the Tb.N in the G/H@AZnBg and G/H@ABg groups were significantly higher than that in the Ctrl and G/H groups (Figure 6C). After 8 weeks, Micro-CT results showed that the BV/TV value of G/H@AZnBg was greater than 70%, indicated higher bone regeneration (Figure 6B). Consistent with the results of Micro-CT, H&E staining showed that G/H@AZnBg significantly promoted bone formation and bone maturation compared with other groups (Figure 7).

**FIGURE 8:**
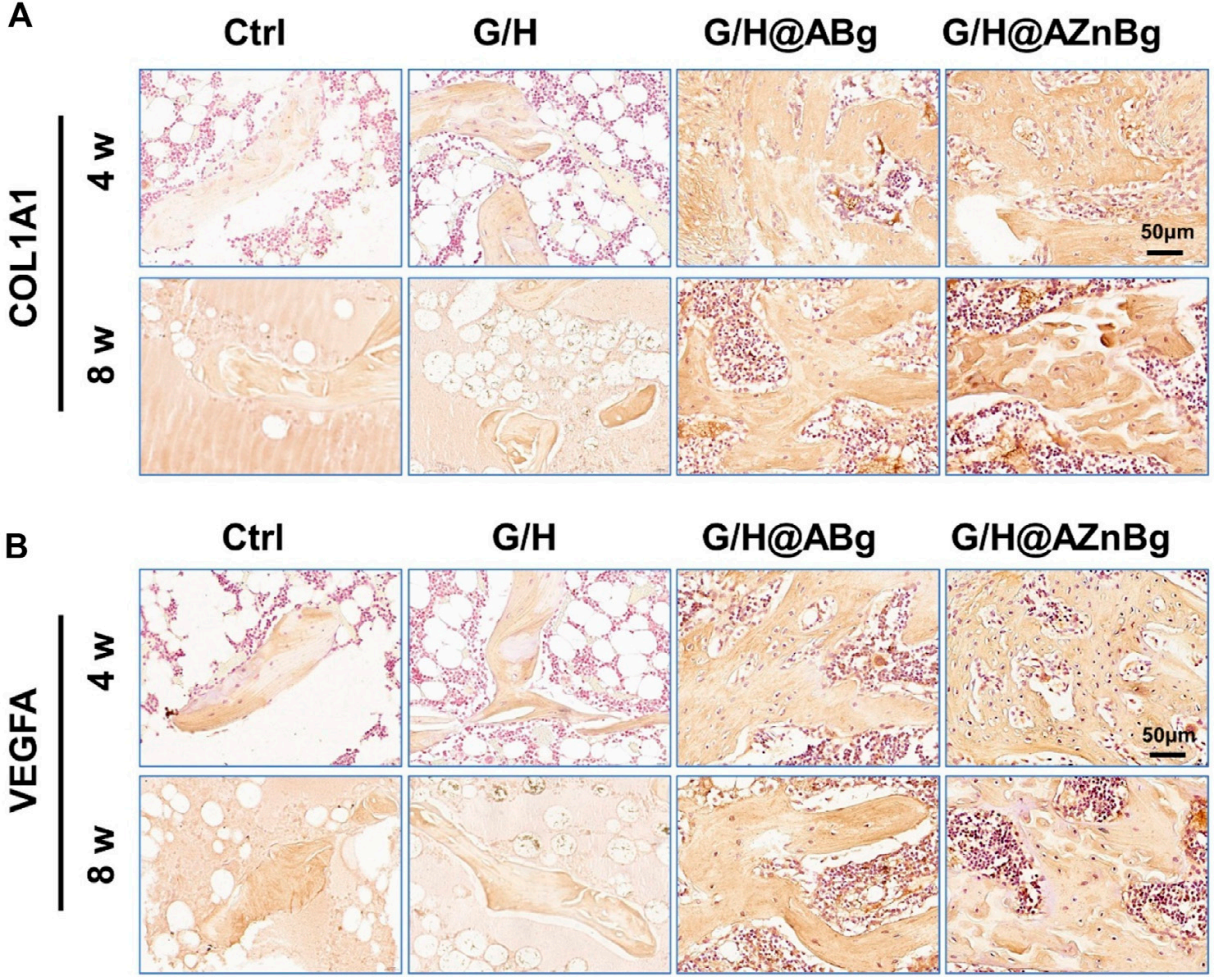
Immunohistochemical analysis of osteogenesis and vascularization. **(A)** Immunohistochemical staining for COL1A1 in the defect area at 4 and 8 weeks post-surgery. **(B)** Immunohistochemical staining for VEGFA in the defect area at 4 and 8 weeks post-surgery.

COL-1 is an important protein for bone formation and its expression reflects the activity of bone formation. As shown in the Figure 8A and Supplementary Figure S4, immunohistochemical staining showed the highest expression of COL1A1 in the G/H@AZnBg group and the lowest expression in the Ctrl and G/H groups. To investigate the effect of G/H@AZnBg on angiogenesis, we performed immunohistochemical staining to detect the expression of VEGFA. Immunohistochemical staining for VEGFA showed the strongest positive expression in the G/H@AZnBg group compared to the other groups (Figure 8B and Supplementary Figure S5). These findings demonstrated the effectiveness of G/H@AZnBg in promoting bone regeneration and facilitating neovascularization, thereby promoting repair of bone defects.

## 4 Discussion

Adequate angiogenesis is essential for the reconstruction and regeneration of the bone matrix during bone repair (Kumar et al., 2022). Neovascularization provides essential nutrients and cytokines for bone regeneration to regulate the process of angiogenesis and osteogenesis (Lee et al., 2021). When the bone defect site is large, biomaterials have limited effect in promoting angiogenesis, which limits their application in tissue engineering. Therefore, improving the angiogenesis of bone repair biomaterials is important to increase the success rate of bone regeneration. In order to promote vascular network generation at defect sites, many scholars have promoted the vascularization of biomaterials by adding growth factors or cytokines (Walsh et al., 2019). Among the growth factors used to stimulate angiogenesis, the most widely used and successful are VEGF, fibroblast growth factors (FGFs), and platelet-derived growth factor (PDGF) (Ribatti and d'Amati, 2023; Cao et al., 2023; Mohan et al., 2022). However, these growth factors have disadvantages such as short duration of action, expensive and not easy to store (Wang et al., 2011). In recent years, doping biomaterials with vasculogenic metal ions has become an effective way to enhance the vasculogenic and osteogenic activities of biomaterials (Ma et al., 2021; Wu et al., 2021). Metal ions such as copper, cobalt, magnesium, zinc and strontium have been shown to have angiogenic activity and are widely used in biomaterials such as hydrogels, composite scaffolds, guided tissue regeneration membranes and other biomaterials for the treatment of various bone-related diseases (Mao et al., 2017; Lai et al., 2019).

Zinc ion is also a relatively rich trace element in the human body, involved in various biological reactions, especially bone metabolism (Raj Preeth et al., 2021; Wen et al., 2023). Some metalloenzymes in the body are regulated and catalyzed by zinc ions, such as ALP, which is closely related to the maturation of new bone. ALP is a glycoprotein containing zinc, which can provide an alkaline environment for the bone regeneration process and promote the mineralization process (Yang et al., 2021). In this study, it was also found that the expression of ALP was significantly promoted in the G/H@AZnBg group compared with the G/H group. In addition, studies in recent years have confirmed the role of zinc ions in promoting angiogenesis, and found that the relationship between angiogenesis and osteogenesis is close (Lin et al., 2019). Song et al. prepared a zinc silicate/nanohydroxyapatite/collagen (ZS/HA/Col) scaffold and found that the scaffold could promote cell secretion of chemokines and VEGF to promote endothelial cell proliferation, migration and angiogenesis (Song et al., 2020). Wang et al. evaluated the effect of magnesium-zinc alloy in enhancing osteogenesis and vascularization, and showed that magnesium and zinc ions showed synergistic effect, which could promote the proliferation of endothelial cells and the neovascularization (Wang et al., 2022). This is consistent with the results of our study, in which the addition of zinc ions significantly promoted angiogenesis and osteogenic differentiation.

Silicon, recognized as an essential trace element crucial for the growth and development of organisms, has been found to facilitate angiogenesis and the establishment of functional vascular networks, regulate bone formation and calcification, and contribute significantly to bone metabolism (Khandmaa et al., 2017a; Huang et al., 2018). The promotion of angiogenesis by silicon ions is achieved through the inhibition of prolyl hydroxylase-2 expression and the upregulation of the HIF-1 α signaling pathway (Khandmaa et al., 2017b). Research has demonstrated that the doping of silicon-coated implants can enhance angiogenesis through the facilitation of adhesion, proliferation, migration, tube formation, and the expression of angiogenesis-related genes in HUVEC (Fu et al., 2020; Monte et al., 2020). Furthermore, it has been observed that strontium-substituted calcium silicate ceramics have the ability to induce bone marrow stromal cells to secrete exosomal miR-146a, thereby modulating osteogenesis and angiogenesis (Liu et al., 2021).

Although zinc ions are more effective in promoting osteogenesis and angiogenesis, excessive zinc ions release rate may inhibit the activity of endothelial cells, thus affecting angiogenesis and osteogenesis (Zhu et al., 2022). To address this issue, in this study, amino-modified zinc-containing bioactive glass was prepared and combined with hydrogels via Schiff base bond. It was found that the composite hydrogel had a good slow-release effect and could release zinc ions slowly and sustainably. Although GelMA has cell adhesion, proliferation, differentiation and other properties conducive to osteogenesis, the lack of mechanical properties limits its application as a load-bearing material. Zheng et al. prepared an organic-inorganic composite hydrogel, which was prepared by physical and chemical cross-linking method using bioactive glass and GelMA, and found that its compression modulus was significantly improved (Zheng et al., 2018). Qian et al. enhanced the osteoinductive properties of PLLA by incorporating Zn-doped mesoporous silica (Zn-MS) particles into the scaffold, resulting in improved osteogenic induction and mechanical characteristics (Qian et al., 2021). Additionally, a separate study demonstrated that the growth of zeolitic imidazolate framework-8 (ZIF-8) on strontium carbonate (SrCO3) to form a core-shell SrCO3@ZIF-8 composite enhanced both the mechanical properties and osteogenic activity of the scaffold (Qian et al., 2024b). These studies show that bioglass and ions can optimize the mechanical properties and osteogenic activity of scaffolds, which are consistent with the results of our study. In addition, the addition of bioactive glass to the hydrogel could significantly improve the stability and bioactivity of the composite hydrogel, which could promote cell attachment, proliferation and osteogenic differentiation. Moreover, the bone microenvironment after injury is often acidic, which is unfavorable to new bone formation. We constructed an acid-responsive hydrogel through Schiff base bonding, which can achieve the purpose of releasing zinc ions in response to acid on the one hand, and improve the acidic microenvironment on the other hand, which is conducive to the formation of new bone.

In our study, we constructed an acid-responsive bone biomaterial by hydrogel-loaded zinc-modified bioglass to improve the bone microenvironment and repair bone defects. Compared with growth factors (Wu et al., 2020; Zhao et al., 2020), incorporating bioactive ions into bone substitutes is a simpler and safer method to promote bone regeneration at a relatively low cost. In addition, different from previous studies (Song et al., 2020; Wang et al., 2022; Tian et al., 2023), we not only used the osteogenic effects of zinc ions, but also used the amino groups in AZnBg and GelMA to form Schiff bases with the aldehyde groups in HA-CHO, thus making the structure of the composite hydrogel more stable and realizing acid-responsive ion release. Through the release of zinc and silicon ions at the site of bone injury, it can improve the angiogenesis and bone induction ability, improve the local microenvironment, and ultimately promote bone regeneration.

## 5 Conclusion

In summary, we have developed a composite hydrogel containing bioglass for repairing bone defects. The zinc-containing bioglass was bonded to the hydrogel via a Schiff base bond, which allowed a sustained and effective slow release of zinc ions. This composite hydrogel has good biocompatibility. In addition, it promoted cell proliferation and facilitated osteogenic differentiation of BMSCs. It is worth noting that the hydrogel can promote angiogenesis by releasing zinc ions. *In vivo* experiment results proved that the composite hydrogel implanted in the bone defect can effectively stimulate the bone formation and the development of new blood vessels, thus accelerating the bone healing process. Therefore, the combination of bioactive glass with zinc ions endows the material with vascularization-promoting and osteoinductive activities, which may provide inspiration for the design of bone repair biomaterials.

## Data Availability

The original contributions presented in the study are included in the article/Supplementary Material, further inquiries can be directed to the corresponding authors.
