# Supplementary material for: Zinc-energized dynamic hydrogel accelerates bone regeneration via potentiating the coupling of angiogenesis and osteogenesis
Source: Front Bioeng Biotechnol. 2024 Apr 3;12:1389397. doi: 10.3389/fbioe.2024.1389397 (PMC11022217; doi:10.3389/fbioe.2024.1389397)
Supplement: Supplementary file 1 [file DataSheet1.docx]

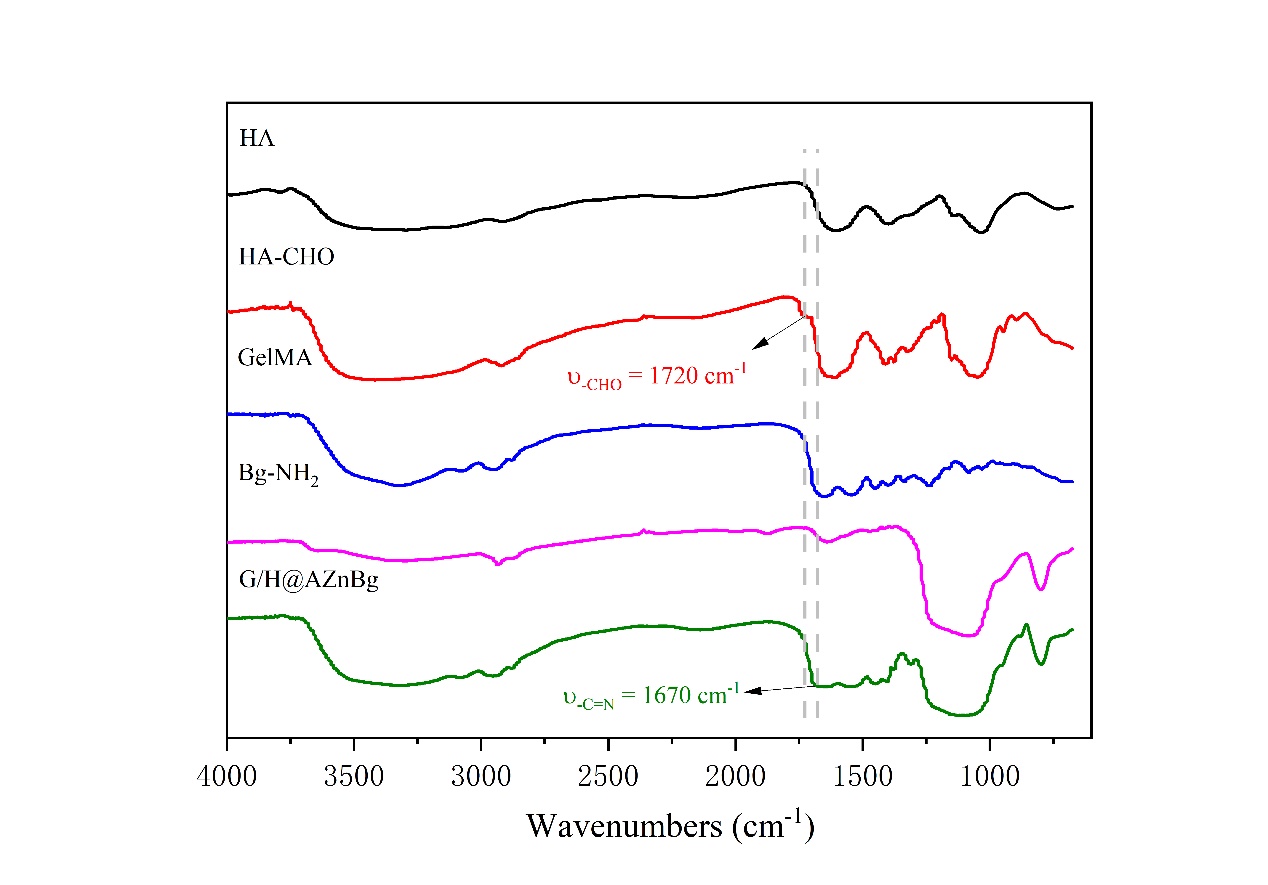


**Supplementary Fig. 1** FTIR spectra of HA, HA-CHO, GelMA, Bg-NH_2_, and G/H@AZnBg.


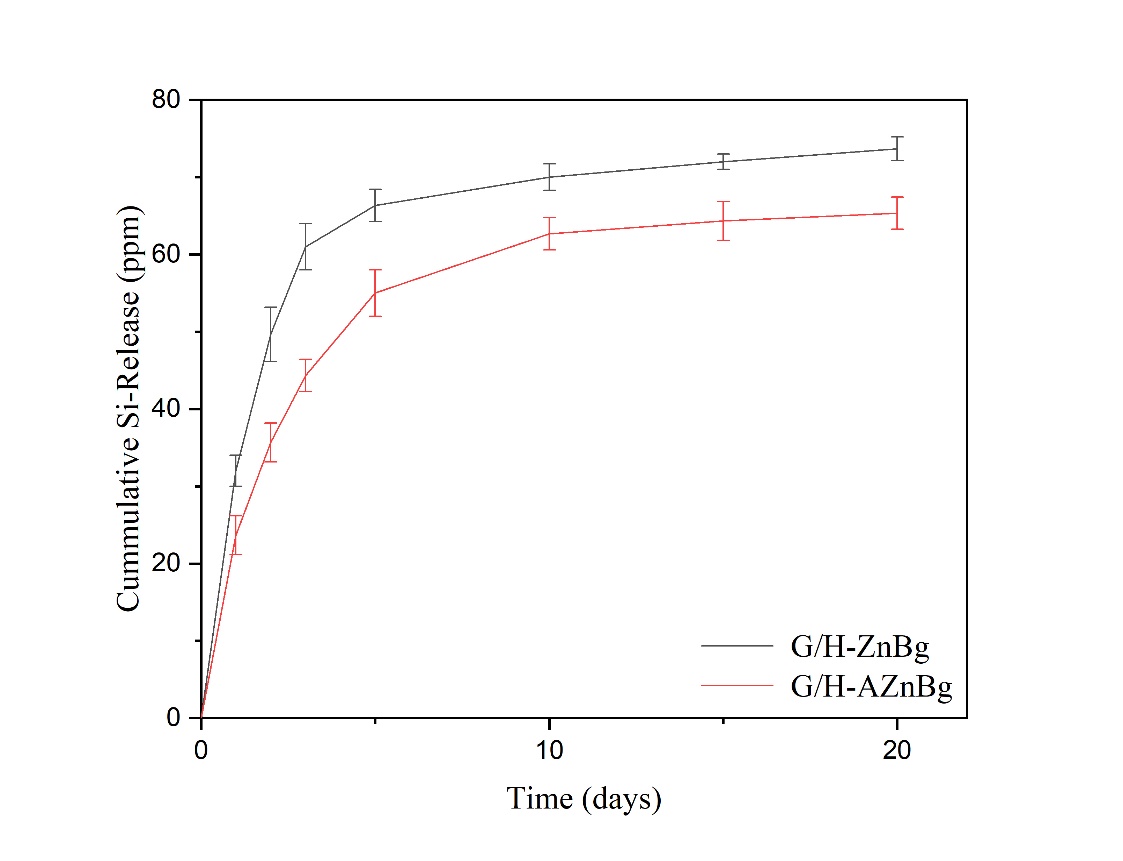


**Supplementary Fig. 2** Release of Si ions from G/H@ZnBg and G/H@AZnBg.


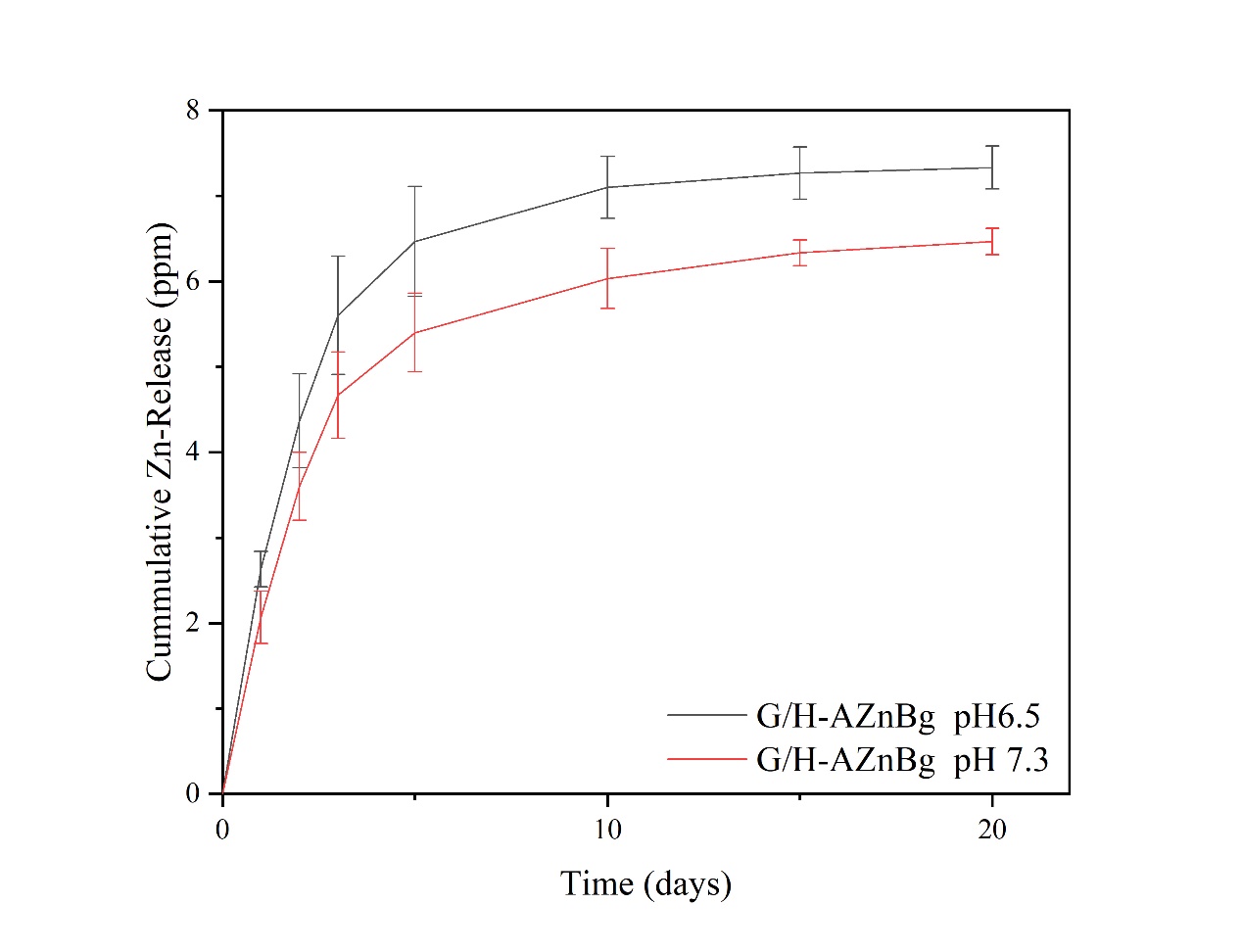


**Supplementary Fig. 3** Release of Zn ions from G/H@ZnBg in pH6.5 and pH7.3


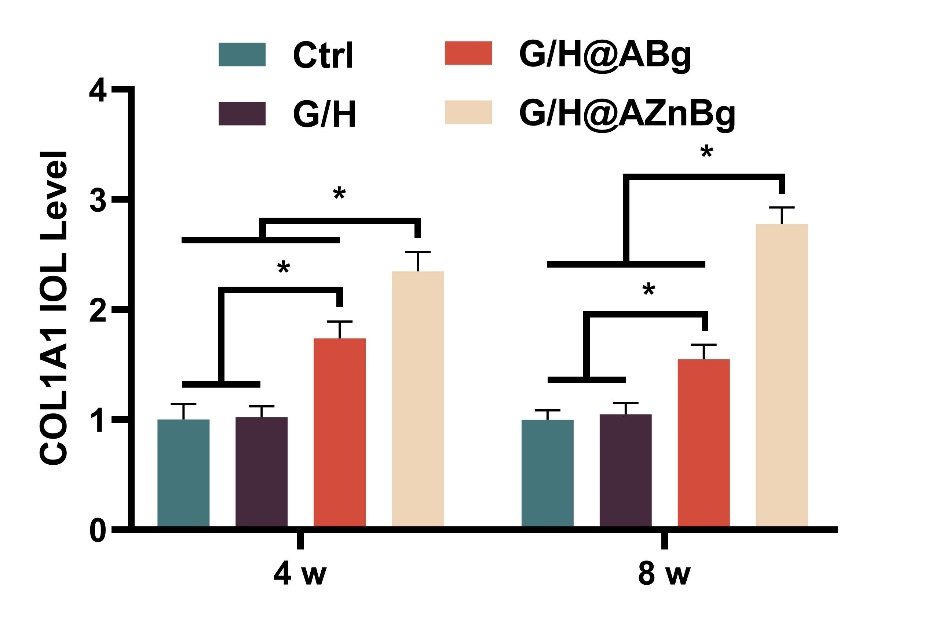


**Supplementary Fig. 4** Quantification of the COL1A1-positive area at 4 and 8 weeks post-surgery.


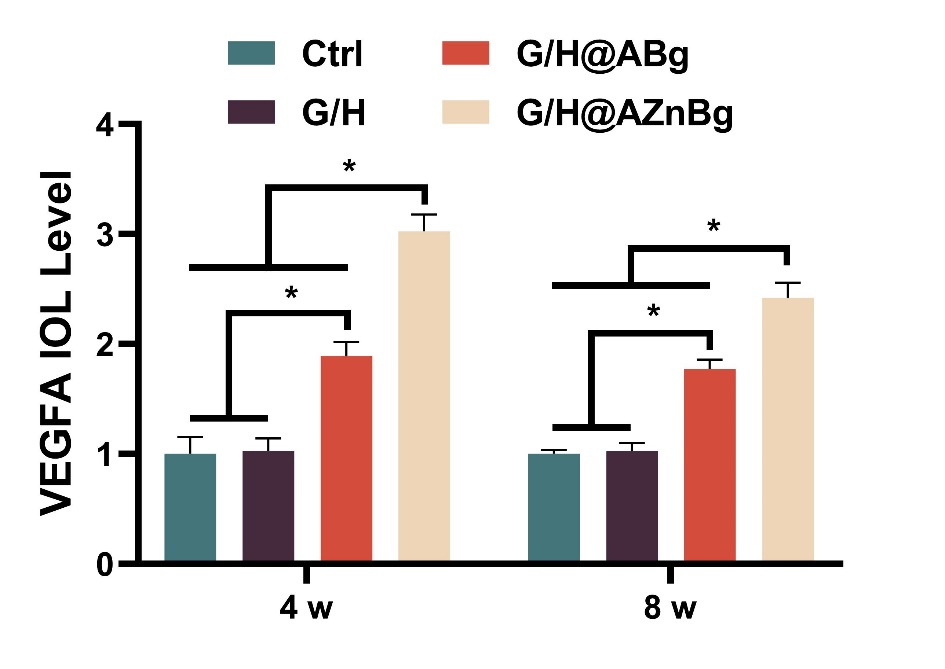


**Supplementary Fig. 5** Quantification of the VEGFA-positive area at 4 and 8 weeks post-surgery.
